# Biomarkers of disease differentiation: HCV recurrence versus acute cellular rejection

**DOI:** 10.1186/1755-1536-5-S1-S11

**Published:** 2012-06-06

**Authors:** Ricardo Gehrau, Valeria Mas, Kellie Archer, Daniel Maluf

**Affiliations:** 1 University of Virginia, Department of Surgery, Transplant Division, P.O. Box 800625, 904 Lane Rd, Charlottesville, VA, 22908-0625, USA; 2 Virginia Commonwealth University, Department of Biostatistics P.O. Box 980032, 730 East Broad Street, Room 3006, Richmond, VA 23298-0032, USA

## Abstract

The wound-healing process induced by chronic hepatitis C virus (HCV) infection triggers liver damage characterized by fibrosis development and finally cirrhosis. Liver Transplantation (LT) is the optimal surgical treatment for HCV-cirrhotic patients at end-stage liver disease. However, acute cellular rejection (ACR) and HCV recurrence disease represent two devastating complications post-LT. The accurate differential diagnosis between both conditions is critical for treatment choice, and similar histological features represent a challenge for pathologists. Moreover, the HCV recurrence disease severity is highly variable post-LT. HCV recurrence disease progression is characterized by an accelerated fibrogenesis process, and almost 30% of those patients develop cirrhosis at 5-years of follow-up. Whole-genome gene expression (WGE) analyses through well-defined oligonucleotide microarray platforms represent a powerful tool for the molecular characterization of biological process. In the present manuscript, the utility of microarray technology is applied for the ACR and HCV-recurrence biological characterization in post-LT liver biopsy samples. Moreover, WGE analysis was performed to identify predictive biomarkers of HCV recurrence severity in formalin-fixed paraffin-embedded liver biopsies prospectively collected.

## Introduction

Hepatic fibrogenesis is considered a model of the wound-healing response to chronic liver injury. This process is characterized by extra-cellular matrix (ECM) proteins accumulation as consequence of an imbalance between the deposition and degradation of ECM components [1-4]. Different stimuli such as cytokines and other extracellular signals, including reactive oxygen species (ROC) produced by parenchymal (mainly stellate cells) and non-parenchymal (mononuclear cellular infiltration) cells have been demonstrated to be involved in the fibrogenesis response to liver injury ([5] and references therein).

**Figure 1:**
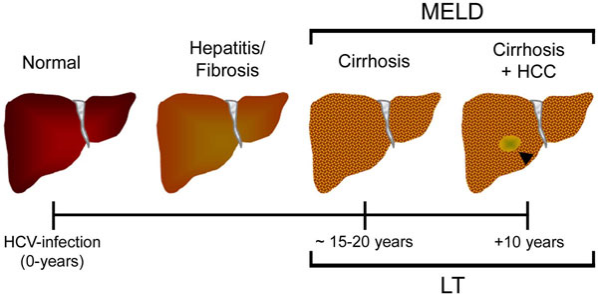
**Schematic representation of the liver wound-healing process in HCV infected patients**. An arrowhead indicates a hepatocellular carcinoma (HCC) lesion. MELD: Model of End Stage Liver Disease. LT: Liver Transplantation.

Hepatotropic viruses, especially hepatitis C (HCV) virus, constitute the principal chronic liver injury etiology [6]. After 15-20 years, most of HCV-chronic infected patients will develop liver cirrhosis characterized by a distortion of the hepatic architecture and vascular structure, and a nodular transformation of the liver surface [1,2,7]. Clinical characteristics of liver fibrosis are associated with hepatocellular dysfunction and increased intrahepatic resistance to blood flow leading to hepatic insufficiency and portal hypertension, and associated hepatocellular carcinoma (HCC) in 5% of cases [8-11]. Despite available therapeutic protocols aimed to treat the etiological agent (virus), stellate cells (involved in liver fibrogenesis), or specific signaling pathways, there is no effective pharmaceutical intervention for liver fibrosis associated with viral injury [5]. In this regard, HCV-cirrhotic patients at end stage liver disease have been prominently beneficiated with liver transplant (LT). Indeed, HCV induced cirrhosis with or without HCC is the leading indication for liver transplantation (LT) in USA, Europe and Japan [12,13] (Figure 1).

Unfortunately, HCV recurrence is universal. HCV RNA serum load dramatically decreases until almost undetectable level within 24-48 hours post-LT. However, it increments few days post-LT with a peak at 1-3 months, and reaching a 1-2 logs plateau higher than the pre-LT viral load after the first year post-LT [14]. Of those patients, about 80-100% will develop HCV recurrence disease, and 25-30% of them will course with accelerated fibrosis progression and concomitant cirrhosis development within 5 years post-LT. These patients require liver re-transplantation or will develop liver failure [13]. In parallel, acute cellular rejection (ACR) represents an additional cause of morbidity and allograft injury in HCV-infected recipients. Despite the rejection risk rate is controversial in HCV patients the incidence round 40% at 6-months post-LT as reported previously by large cohort studies [15]. Both post-LT complications have clinical and histopathological overlapped features turning difficult the accurate differential diagnosis, which may threat the allograft and patient survival rate due to the opposite therapy [15-17].

The discovery of reliable biomarkers for differential diagnosis of both complications, and also for HCV recurrence disease surveillance constitutes the research endeavor. The implementation of whole-genome gene expression (WGE) studies using microarray technology represents an outstanding opportunity for biomarkers discovery. The present article is focused on performed WGE analysis aimed to differentiate ACR in the setting of HCV recurrence disease, and to predict liver fibrosis progression in HCV-infected recipients.

### ACR and HCV recurrence differential diagnosis post-LT: the paradigm

The differential diagnosis of ACR in the setting of HCV recurrence disease remains an important cause of morbidity and late graft failure in liver-transplant recipients. The assessment of liver allograft biopsy is still considered to be the "*gold standard*" for proper differentiation between both post-LT conditions. However, subtle similarities in the histopathology and clinical course turn difficult and uncertain the pathological differentiation, even among experiences hepatologists [18,19]. HCV recurrence disease have been described in three major forms: Acute or chronic recurrence and the less frequent and more aggressive primary cholestatic disease called fibrosing cholestatic hepatitis. All of these forms course with a characteristic lobular inflammatory cellular infiltrate [20]. ACR occurs due to the attacks of the recipient immune system to the allograft and it is characterized by severe inflammatory infiltrate of the portal tract with the bile duct epithelial cells and the endothelium of hepatic arteries and veins as major targets. The appropriate clinical differentiation between both conditions directly impact in the associated therapy decision basically by the administration of steroid bolus for ACR treatment. Unfortunately, the ACR therapy results contradictory for HCV recurrence cases due to the induction of an exacerbation of the HCV infection accompanied by worse allograft and patient survival [16,21].

The implementation of large-scale genomic analyses strategies has provided new insights into various disease processes and had assisted on elucidating genomic patterns for mechanism, diagnosis, prognosis, and treatment selection of complex and multi-factorial diseases [13]. For instance, microarray technology represented a powerful tool for the molecular understanding and knowledge of involved gene networks and regulatory pathways for each particular condition.

**Figure 2:**
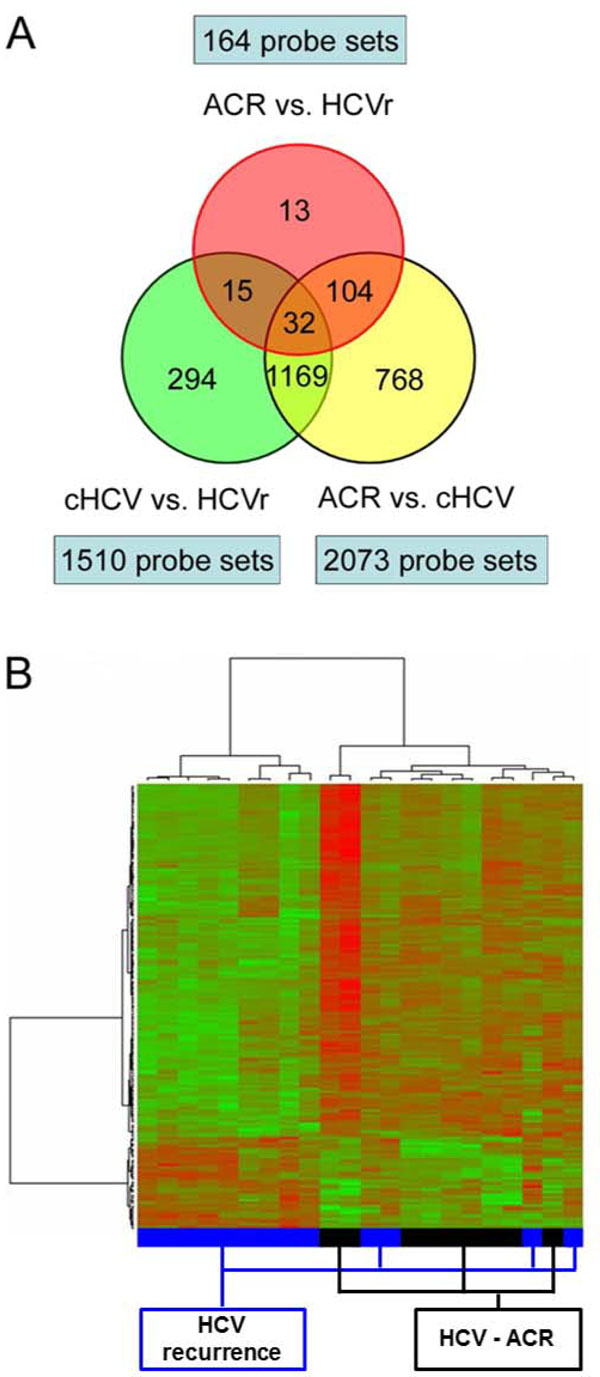
**Molecular characterization of HCV-infected liver biopsy samples diagnosed with ACR or HCV recurrence**. **A**. Venn diagram illustrating a total of 3747 differentially expressed probesets in three pairwise comparisons composed by HCV-ACR (acute cellular rejection in HCV infected patients), HCV recurrence, and chronic HCV liver biopsy samples. **B**. Heatmap and dendrogram resulting from agglomerative hierarchical clustering using Ward's method, using 1-Pearson's correlation for distance measure.

As a first approach, differentially expressed genes were evaluated in ACR and HCV recurrence liver biopsy samples from HCV-infected recipients using oligonucleotide GeneChip^® ^(Affymetrix Inc., CA, USA). The study was approved by the Institutional Review Board at Virginia Commonwealth University, and informed consents were obtained from all patients. The patients cohort included liver biopsy samples from 24 HCV-recipients with histological diagnosis of HCV recurrence disease (n = 13) and ACR in the setting of HCV (HCV-ACR; n = 11). Liver tissue from chronic HCV-infected patients without LT (Chronic-HCV; n = 10) were included for comparison analysis proposes. Tissue collection, total RNA isolation and quality control criteria, cDNA synthesis, in vitro transcription for labeled cRNA probe, and microarray hybridization and analysis were performed as described previously [22]. No significant differences in patient age and histological inflammatory activity index were identified between groups. All patients were Caucasian, male and infected with HCV genotype 1b. From the analysis, 3747 probesets were found to be significantly differentially expressed among three pairwise comparisons (HCV-ACR vs. HCV recurrence; HCV-ACR vs. Chronic-HCV; Chronic-HCV vs. HCV recurrence). Of those, 164 probesets were found to be differentially expressed between HCV-ACR and HCV-recurrence alone samples. Thirteen probe sets were found unique for both conditions (Figure 2A). Gene ontology and gene interaction analyses were performed using Ingenuity Pathways Analysis tools http://www.ingenuity.com. Those differentially expressed genes in ACR biopsy samples were associated with pathways involved in immune and inflammatory responses, apoptosis, complement system, and growth factor receptors. HCV recurrence samples revealed predominantly gene expression patterns associated with cell cycle and cell division, cell proliferation and blood coagulation. The supervised hierarchical clustering analysis including specific probesets differentially expressed for HCV-ACR *vs*. HCV recurrence clustered all samples in two well-differentiated groups characterizing both different post-LT conditions (Figure 2B). This first analysis clearly demonstrates the potential efficacy of gene expression analysis to differentiate ACR from HCV recurrence post-LT [13].

**Figure 3:**
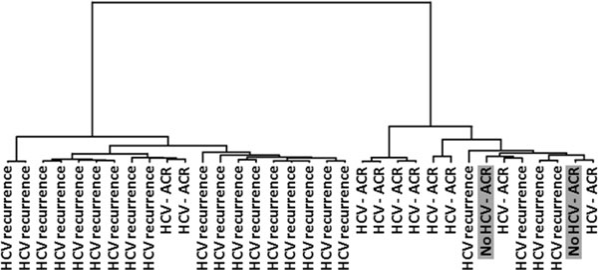
**Supervised hierarchical clustering for HCV-ACR, ACR-alone, and HCV recurrence diagnosed liver biopsy samples**. The dendrogram illustrates clustered samples using Ward's method when a total of 80 differentially expressed probesets are included. ACR samples from no-HCV-infected recipients are highlighted in grey boxes.

In a second approach, a similar analysis was performed by increasing the number of samples. A total of 51 biopsy samples from unique HCV-infected recipients were included in the analysis. A training set composed by 32 ACR liver samples from HCV-infected recipients and 2 ACR liver samples from no HCV-infected recipients (n =34), were evaluated using microarray techniques. From the analysis, 179 probesets were found significantly differentially expressed among HCV-ACR, no-HCV-ACR, and HCV recurrence biopsy samples. A total of 71 exclusive genes were identified for HCV-ACR vs. HCV recurrence pairwise comparison. Interestingly, no significant probesets were found differentially expressed between ACR samples from HCV and no-HCV infected recipients, which suggest a particular ACR molecular nature independent of the HCV infection. Gene ontology analysis identified canonical pathways specifically associated to a cytotoxic T cell profile in for HCV recurrence samples, and inflammatory response related genes as the ACR profile. The supervised clustering analysis including probesets (n = 80) representing those 71 specific genes displayed two well-differentiated groups for ACR and HCV recurrence samples. Interestingly, no-HCV-ACR samples were clustered within the HCV-ACR group (Figure 3).

In parallel, a LASSO model was fit with HCV recurrence vs. HCV-ACR as the dependent viable predicted, followed of N-fold cross validation to provide an unbiased estimate of generalization error. Interestingly, the best fitting-LASSO model included 15 genes with an accuracy of 100% for the training set, while the N-fold cross-validation accuracy was of 78.1%. Four out of those fifteen genes were also included into the exclusive gene list, and further validated in an independent set of 19 biopsy samples (validation set) [23].

The concluding results from the analysis of both experimental approaches using GeneChip^® ^microarrays clearly demonstrate a differential gene expression signature between both conditions. Indeed, HCV recurrence disease is characterized by an adhesion and apoptosis of cytotoxic T cells profile regulated by canonical pathways related to IFN-γ and NFκB, while ACR is related to genes associated with an immediate hypersensitivity reaction [23]. The histopathological features of both conditions are related with mottled hepatocytes apoptosis with a Th-1 type profiled lobular inflammation for HCV recurrence disease, and inflammation of the portal tract, bile ducts and hepatic vessel endothelium characterized by CD4^+ ^and CD8^+ ^T cells together with macrophages and eosinophil cells for ACR [16,24,25]. Thus, the genomic profiles identified by this study correlate well with the previously described cellular population that characterized both conditions [13,23]. More importantly, it demonstrates the possibility to establish specific biomarker panels to be combined with the conventional histopathological assessment.

### Prediction of fibrosis progression in HCV recurrence disease

Chronic HCV-related hepatic insufficiency is associated with decreased rates of patient and allograft survival in comparison with other indications for liver transplantation [26]. Different factors inherent to the donor, recipient, and post-transplant variables have been associated with progression and severity of the liver allograft injury course in HCV recurrence disease [13,27]. Nowadays any of those factors were established as independent predictors of HCV recurrence severity at early stage of the disease in HCV-infected recipients. For instance, it became critical the identification of reliable biomarker predictors of disease progression at the time of HCV recurrence diagnosis since the high variable aggressiveness of the disease.

**Figure 4:**
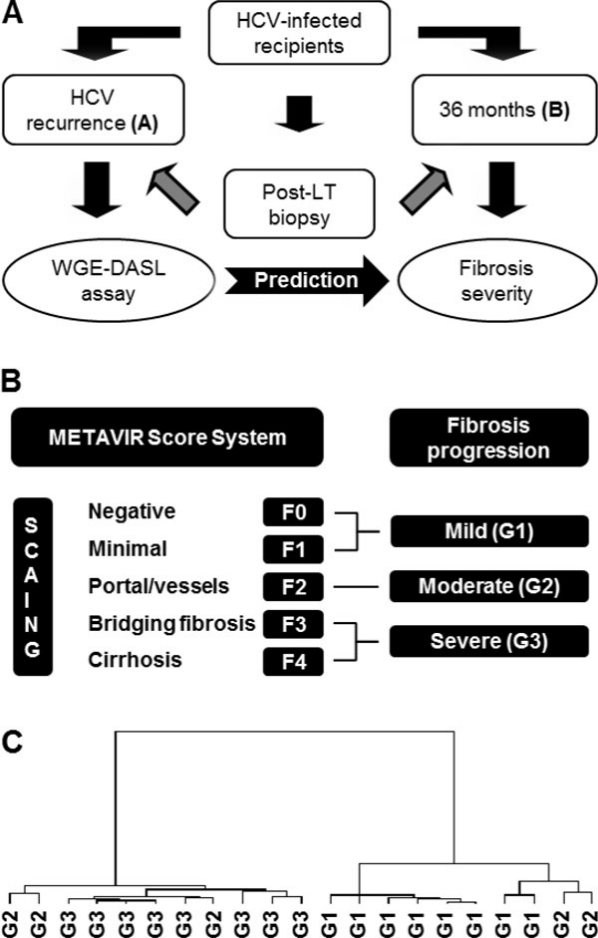
**Molecular characterization of HCV recurrence samples at diagnosis time for disease severity progression prediction**. **A**. Schematic illustration of a retrospective analysis using prospectively collected FFPE liver biopsy samples at HCV recurrence diagnosis (A) and 3-years of follow-up (B) as time-point for classification. **B**. METAVIR score criteria used for the classification of FFPE liver biopsy samples. Liver biopsy samples at HCV recurrence diagnosis time were classified in three groups (G1, G2, and G3) depending on the fibrosis severity of paired 3-years follow-up biopsy samples. **C**. Dendrogram illustrating a supervised agglomerative hierarchical clustering with 50 differentially expressed beads identified between G1 vs. G3 using DASL assay.

Molecular profiling at early stages of the disease might provide important information regarding mechanisms involved in accelerated fibrogenesis progression as HCV recurrence severity indicator. Even more, analysis of historical liver biopsy samples represents a feasible research scenario. In this regard, genome-wide gene expression analysis has been performed in Formalin-Fixed Paraffin-Embedded (FFPE) liver biopsies at the time of HCV recurrence diagnosis [13,28]. HCV recurrence was defined as post-transplant increased serum level of Alanine Aminotransferase (ALT) and positive PCR for HCV as previously shown [28]. Progression of the disease was determined by the fibrosis severity in FFPE biopsy samples at 36 months post-LT (Figure 4A). Fibrosis stages were determined using METAVIR score system [29]. A total of 42 unique samples from 21 adult patients who underwent LT between 1995 and 2006 were included. Samples at HCV recurrence diagnosis time were grouped as Mild (G1; n = 8), Intermediate (G2; n = 5), and Severe (G3; n = 8) referred to the follow-up biopsy at 3-years post-LT (Figure 4B). The study population were composed by 25 (58%) and 18 (42%) white male and female HCV recipients. All patients did not receive HCV treatment, and the mean time from LT to HCV recurrence was 5.5 ± months. The predominant HCV genotype were 1 (84%), and 7 patients were infected with genotypes 2 or 3. Total RNA was isolated using Recover All™ Total Nucleic Acid Isolation Kit (Ambion, Austin, TX, USA). Quality control parameters for required RNA purity for WGE analysis have been established and tested [28]. Differential gene expression analysis among study groups were performed using WGE-DASL^® ^Assay following manual instructions (Illumina Inc., USA). HumanRef-8 Expression BeadChips (Illumina, Inc., USA) hybridization and followed analyses were described in Mas et al 2011 [28]. From the analysis, a total of 57 bead types were found to be significant (*p *≤ 0.001) and differentially expressed after a moderate F test statistical assay. By linear contrast examination, fourteen beads were found between G1 vs. G2, five beads between G2 vs. G3, and fifty beads between G1 vs. G3. Agglomerative hierarchical clustering analysis was performed by incorporating only the 50 beads differentially expressed between G1 vs. G3. The analysis displayed two independent groups composed by G1 or G3 samples. Interestingly, G2 samples were found to be randomly associated within both clusters may be reflecting a pathology miss-classification component (Figure 4C). Gene ontology and gene pathway analysis, using only those 50 beads, identified 9 gene-associated networks, whereas the top-scored was involved in cellular development, infection mechanism, and antigen presentation. The molecular and cellular function analysis associated those beads with cell-to-cell signaling and interaction, cell death, cell morphology, and carbohydrates metabolism. Interestingly, genes associated with T cells biology such as IL-28RA, IL-28, suppressor of T cell receptor signaling 1 (STS1), and CAP-GLY domain containing linker protein 4 (CLIP4) were found to be significantly increased in samples with predicted severe fibrosis [28].

Yet, the mechanisms involved in HCV recurrence development and progression are largely unknown. WGE analyses demonstrated to be a useful tool to either reveal the HCV recurrence molecular biology, and to encourage the identification of potential biomarkers to predict disease severity. A set of no invasive biomarkers have been proposed with promising results for the hepatic fibrosis progression assessment using specific peripheral blood serum proteins as biomarkers [30-32]. Up today, the established protein assay test demonstrated excellent utility for the identification of HCV-advanced cirrhosis, but slighter accuracy for earlier stages of the disease [32]. Thus, only large cohort prospective studies in will contribute to optimize the analytical performance of those tests. At meantime, liver biopsy remains to be the gold standard for allograft fibrosis progression assessment in HCV recurrence despite its well-known no perfect accuracy [33-36]. The combined molecular markers detection and pathology characterization, together with reliable clinical data collected during the liver biopsy protocol may help to predict the severity of HCV recurrence to come.

### Overall comments

The accurate follow-up and the differential diagnosis of post-transplant complications have been further struggled by no reliable and difficult pathology reports, essentially in post-transplanted HCV-infected patients [18]. However, the implementation of WGE analyses permitted new insights about the molecular biology characterization of certain post-LT complications [13,23,28,37,38]. Furthermore, the extension of this technique to the follow-up of HCV recurrence patients might permit the diagnosis and surveillance of severity in the fibrosis progression, along with pathological evaluation [28]. From this perspective, it is imperative the continuous study of different molecular aspects of mechanisms involved in HCV infection and ACR. The complete understanding of the biological process triggered in each condition will allow the identification of early predictors for disease differentiation and progression, and the implementation of them to the diagnostic arsenal. Importantly, it will impact directly in the identification and treatment success rates.

## List of abbreviations used

ACR: Acute cellular rejection; ALT: Alanine aminotransferase; cDNA: copy Deoxyribonucleotide acid; CLIP4: CAP-GLY domain containing linker protein 4; cRNA: copy Ribonucleotide acid; DASL: cDNA-mediated Annealing, Selection, Extension, and Ligation; ECM: Extra-cellular matrix; FFPE: Formalin-fixed paraffin embedded; HCC: Hepatocellular carcinoma; HCV: Hepatitis C virus; IFN-γ: Interferon-gamma; IL-28: Interleukin-28; IL-28RA: Interleukin-28 receptor, alpha (interferon, lambda receptor); LASSO: Least absolute shrinkage and selection operator; LT: Liver transplant; MELD: Model of end-stage liver disease; NFκB: Nuclear factor kappa-B; RNA: Ribonucleotide acid; ROC: Reactive oxygen species; STS1: Suppressor of T cell receptor signaling 1; WGE: Whole-genome gene expression.

## Competing interests

The authors declare that they have no completing interests, or other interests that might be perceived to influence the content included into this article.
